# Abrupt involution induces inflammation, estrogenic signaling, and hyperplasia linking lack of breastfeeding with increased risk of breast cancer

**DOI:** 10.1186/s13058-019-1163-7

**Published:** 2019-07-17

**Authors:** Mustafa M. Basree, Neelam Shinde, Christopher Koivisto, Maria Cuitino, Raleigh Kladney, Jianying Zhang, Julie Stephens, Marilly Palettas, Allen Zhang, Hee Kyung Kim, Santiago Acero-Bedoya, Anthony Trimboli, Daniel G. Stover, Thomas Ludwig, Ramesh Ganju, Daniel Weng, Peter Shields, Jo Freudenheim, Gustavo W. Leone, Gina M. Sizemore, Sarmila Majumder, Bhuvaneswari Ramaswamy

**Affiliations:** 1 The Comprehensive Cancer Center, College of Medicine, The Ohio State University, 460 West 12th Avenue, Columbus, OH 43210 USA; 2 Hollings Cancer Center, Medical University of South Carolina, Charleston, SC USA; 3 Department of Biochemistry & Molecular Biology, Medical University of South Carolina, Charleston, SC USA; 4 Department of Biomedical Informatics’ Center for Biostatistics, The Ohio State University, Columbus, OH USA; 5 Department of Internal Medicine, College of Medicine, The Ohio State University, 320 West 10th Avenue, Columbus, OH 43210 USA; 6 Department of Pathology, The Ohio State University, Columbus, OH USA; 7 Department of Radiation Oncology, The Ohio State University, Columbus, OH USA; 8 Department of Epidemiology and Environmental Health, University at Buffalo, Buffalo, USA

**Keywords:** Breastfeeding, Breast cancer, Abrupt involution, Luminal progenitor cells, Collagen

## Abstract

**Background:**

A large collaborative analysis of data from 47 epidemiological studies concluded that longer duration of breastfeeding reduces the risk of developing breast cancer. Despite the strong epidemiological evidence, the molecular mechanisms linking prolonged breastfeeding to decreased risk of breast cancer remain poorly understood.

**Methods:**

We modeled two types of breastfeeding behaviors in wild type FVB/N mice: (1) normal or gradual involution of breast tissue following prolonged breastfeeding and (2) forced or abrupt involution following short-term breastfeeding. To accomplish this, pups were gradually weaned between 28 and 31 days (gradual involution) or abruptly at 7 days postpartum (abrupt involution). Mammary glands were examined for histological changes, proliferation, and inflammatory markers by immunohistochemistry. Fluorescence-activated cell sorting was used to quantify mammary epithelial subpopulations. Gene set enrichment analysis was used to analyze gene expression data from mouse mammary luminal progenitor cells. Similar analysis was done using gene expression data generated from human breast samples obtained from parous women enrolled on a tissue collection study, OSU-2011C0094, and were undergoing reduction mammoplasty without history of breast cancer.

**Results:**

Mammary glands from mice that underwent abrupt involution exhibited denser stroma, altered collagen composition, higher inflammation and proliferation, increased estrogen receptor α and progesterone receptor expression compared to those that underwent gradual involution. Importantly, when aged to 4 months postpartum, mice that were in the abrupt involution cohort developed ductal hyperplasia and squamous metaplasia. Abrupt involution also resulted in a significant expansion of the luminal progenitor cell compartment associated with enrichment of Notch and estrogen signaling pathway genes. Breast tissues obtained from healthy women who breastfed for < 6 months vs ≥ 6 months showed significant enrichment of Notch signaling pathway genes, along with a trend for enrichment for luminal progenitor gene signature similar to what is observed in *BRCA1* mutation carriers and basal-like breast tumors.

**Conclusions:**

We report here for the first time that forced or abrupt involution of the mammary glands following pregnancy and lack of breastfeeding results in expansion of luminal progenitor cells, higher inflammation, proliferation, and ductal hyperplasia, a known risk factor for developing breast cancer.

**Electronic supplementary material:**

The online version of this article (10.1186/s13058-019-1163-7) contains supplementary material, which is available to authorized users.

## Introduction

Global epidemiological studies link prolonged breastfeeding with a decreased risk of developing breast cancer, in particular, triple-negative breast cancer (TNBC), an aggressive cancer subtype with high mortality [1–9]. In fact, it is estimated that breastfeeding prevents 20,000 breast cancer deaths annually based on the current breastfeeding rates [10]. Yet, a study conducted by our group showed that only 16% of mothers received the information regarding this personal health benefit from breastfeeding from health care professionals [11]. Epidemiological data also show that African-American women have one of the lowest breastfeeding rates in the USA (33% compared to 63% for non-Hispanic Caucasian women) and have a disproportionate burden of developing TNBC, in particular basal-like breast cancer (BLBC) [12]. The Breast Cancer Etiology in Minorities (BEM) study showed that the TNBC risk was increased more than twofold for women with high parity (≥ 3) and no or short-term breastfeeding. The same study showed that when compared to nulliparous women, those who breastfed ≥ 24 months over their lifetime had no increased risk [13]. Additionally, even in women with *BRCA1* and *BRCA2* mutations who have an increased risk of developing TNBC [14–16], breastfeeding for more than a year reduces this risk by 32%, reiterating the potential impact of prolonged breastfeeding on prevention [17].

The mechanism by which breastfeeding affects breast cancer development is unclear. The human pregnancy-lactation-involution cycle is a dynamic, multi-step process. During pregnancy, the mammary epithelium undergoes extensive proliferation and further differentiates during lactation to produce milk. At the post-lactation involution stage, the bulk of the mammary epithelium undergoes programmed cell death, while a small fraction of the cells remodels to a pre-pregnancy state [18]. However, the remodeling process varies based upon the length of breastfeeding. When breastfeeding is not initiated after birth or is stopped abruptly within a short time after initiation, the breast tissue undergoes forced and abrupt remodeling (abrupt involution (AI)). When breastfeeding occurs over a long period and ends gradually as infants wean off their mothers’ milk, the breast tissue remodels gradually over time (gradual involution (GI)). While the molecular changes associated with mammary gland involution following pregnancy and lactation have been studied in murine models, these studies have used a model in which pups were weaned abruptly on postpartum days 10–14, simulating the effects of AI but not GI [19, 20]. To date, the molecular changes occurring during AI have not been compared to changes occurring in mammary glands that have undergone gradual involution following prolonged lactation and gradual weaning. Given that gradual involution of mammary glands is epidemiologically linked to decreased risk of breast cancer, this is an important area of continued research.

We present here for the first time results of our study identifying distinct histological and molecular changes in mammary glands from mice undergoing AI compared to those undergoing GI. We used FVB/N mice to model abrupt and gradual involution and found higher proliferative and inflammatory markers and collagen deposition in the AI cohort. Notably, glands undergoing AI subsequently developed ductal hyperplasia and squamous metaplasia. AI glands also demonstrated persistent expansion of the mammary luminal progenitor (LP) cell population, the putative cell of origin of *BRCA1*-associated and sporadic BLBC [21, 22]. Gene set enrichment analysis (GSEA) of mammary LP cells obtained from AI mice revealed distinct enrichment of pathways important in stem cell maintenance and cell survival, an observation also demonstrated in breast tissue obtained from parous women with no history of breast cancer who breastfed cumulatively for less than 6 months. This was not observed in women who breastfed longer. Combined, we demonstrate a potential direct biological link between the lack of breastfeeding and increased future risk of developing TN/BLBC.

## Materials and methods

### Mouse model

**Fig. 1:**
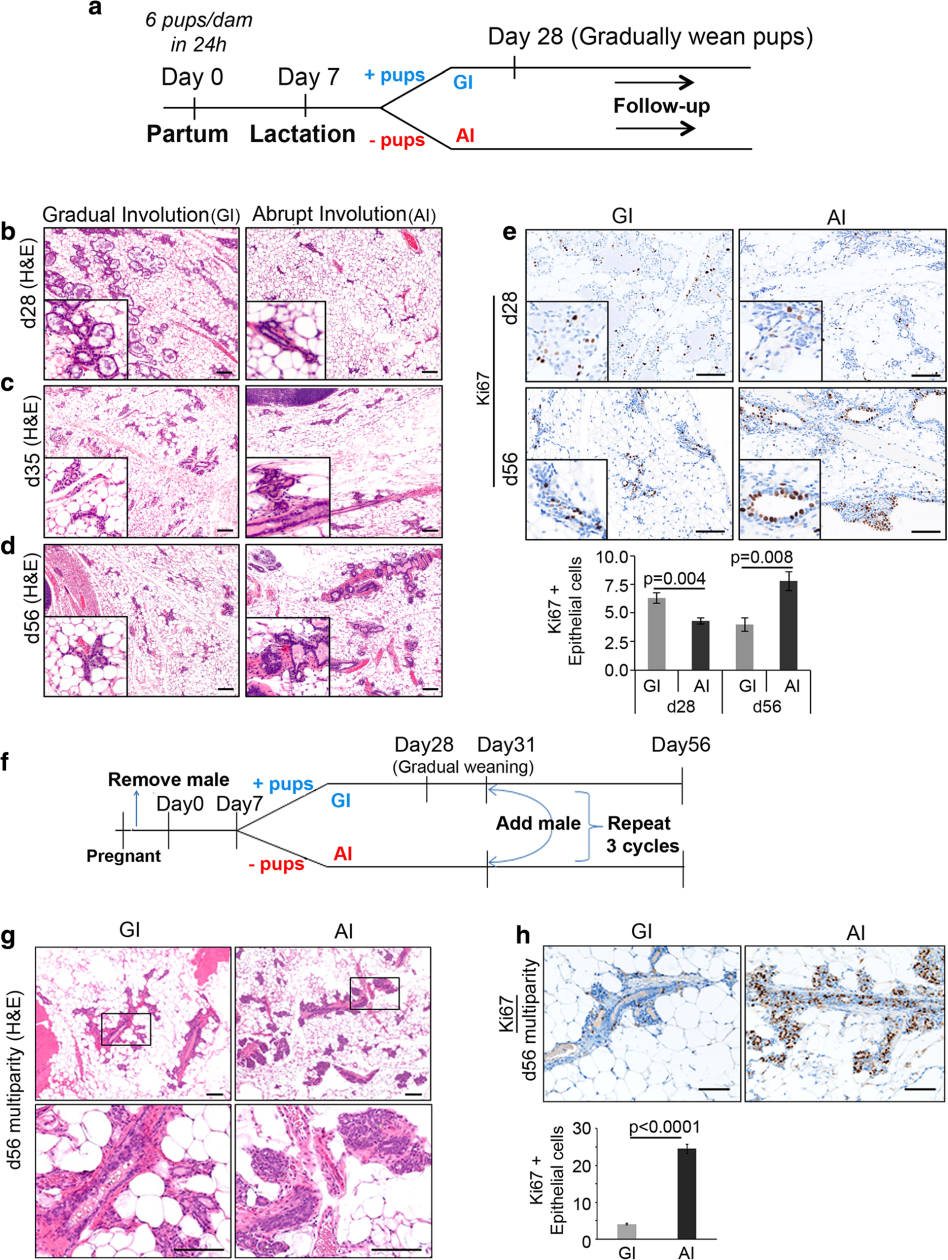
Mouse mammary glands undergoing abrupt involution are histologically different from gradually involuted glands. **a** Experimental workflow for modeling pregnancy, lactation, and involution in mice. GI gradual involution, AI abrupt involution. **b** Representative images of H&E-stained mammary gland sections harvested on day 28 (d28), **c** day 35 (d35), and **d** day 56 (d56) postpartum, following GI and AI. High-magnification pictures are shown in the inset. (*n* ≥ 3, scale bar = 100 μM) **e** Representative images of Ki67-immunostained sections of mammary glands harvested on d28 and d56 following GI and AI (*n* = 3, scale bar = 100 μM). % Ki67-positive epithelial cells are represented in the bar diagram. **f** Experimental workflow for modeling multiparity, lactation, and involution in mice. **g** Representative images of H&E-stained mammary gland sections from GI and AI mice harvested on day 56 postpartum following third pregnancy. The lower panel shows high-magnification picture of the boxed region in the upper panel (*n* = 4, scale bar = 100 μM). **h** Representative images of Ki67-immunostained sections of mammary glands from multiparous mice harvested on d56 following third pregnancy (*n* = 4, scale bar = 100 μM). % Ki67-positive epithelial cells are represented in the bar diagram. Error bars represent standard error of the mean. The linear mixed model was used to calculate significance

All mice experiments were conducted in accordance with a protocol approved by The Ohio State University, University Laboratory Animal Resources, and Institutional Animals Care and Use Committee. All mice were of FVB/N genetic background (Jackson Laboratories, USA, strain# 001800) maintained in-house in barrier cages, under aseptic conditions, and given food and water ad libitum. Post-pubertal virgin mice (8 weeks old, parous group) were mated once and housed individually. Within 24 h postpartum, litter size was standardized to 6 pups. At 7 days postpartum, mice were randomized to either GI or AI cohort. For the AI cohort, all pups were removed on postpartum day 7 reflecting short-term breastfeeding and abrupt weaning in humans. For the GI cohort, 3 pups were weaned on postnatal day 28 and the last 3 pups on day 31 reflecting prolonged breastfeeding and gradual weaning that happens in humans (Fig. 1a). Mice were euthanized on day 28, day 35, day 56, or day120 postpartum following single parity, and mammary glands were harvested for further analysis. For the multiparity experiment, all the females went through three rounds of pregnancies. After the first pregnancy, all the females were paired again with males on day 31 postpartum and were assigned the same group (GI or AI) as for the first pregnancy and the same schema of pup removal for GI and AI cohorts were followed as described (Fig. 1f). All the mice subjected to multiparity were euthanized on day 56 postpartum, following the third pregnancy considering the day of third partum as day 0. Age-matched virgin nulliparous mice were used as controls. Experimental animals were humanely euthanized by CO_2_ inhalation followed by cervical dislocation before necropsy.

### Mammary gland whole mount

Inguinal mammary glands were harvested and fixed in Carnoy solution (60% ethanol, 30% chloroform, and 10% acetic acid) overnight at room temperature, followed by staining with Carmine alum solution (0.2% carmine, 0.5% potassium aluminum sulfate) overnight. Glands were then sequentially dehydrated in graded alcohol, cleared, and stored in xylenes. Cleared glands were mounted using Cytoseal XYL and imaged by a Zeiss Stemi SV11 microscope using Zen software (Zeiss).

### Histological analysis and immunohistochemistry (IHC)

Mammary glands were fixed in a 10% neutral-buffered formalin solution for 48 h following harvest and stored in 70% ethanol. Formalin-fixed, paraffin-embedded (FFPE) sections were stained with hematoxylin and eosin (H&E) for histological analysis. Hyperplastic and metaplastic changes in mammary gland sections were evaluated by blinded histopathologists. Specifically, H&E sections were visually inspected for cell shape, overall cellularity, mitotic figures, and abundance of alveolar structures. All sections were immunostained with Ki67 to confirm hyperplasia.

Immunostaining of the FFPE sections was performed using Bond Rx autostainer (Leica). Primary antibodies used for IHC were anti-Ki67 (1:200, Abcam ab16667), anti-pStat3(Y705) (1:100, Cell Signaling Technology #9145), anti-ERα (1:2000, Abcam ab32063), anti-PR (1:400, ThermoFisher RM9102S0), anti-CD3 (1:100, Abcam ab16669), anti-αSMA (1:5000, Abcam ab124964), anti-CD45R/B220 (1:300 BD Pharmingen #550286), and anti-F4/80 (1:500 Invitrogen MF48000). All the mice were estrous cycle staged, and mice at diestrous were excluded from our analysis.

### Masson trichrome stain

FFPE tissue sections were treated and rinsed sequentially with Bouin’s solution (Sigma), Weigert’s iron hematoxylin A and B mixture (Electron Microscopy Sciences), Biebrich Scarlet-Acid Fusion (Sigma), phosphomolybdic/phosphotungstic acid solution (Sigma), and aniline blue (Sigma). Sections were then dehydrated, cleared, and mounted using synthetic resin. Stained sections were imaged using Vectra 3.0 Automated Quantitative Pathology Imaging System.

### PicroSirius Red stain

Deparaffinized, cleared, and hydrated FFPE tissue sections were incubated in Weigert’s iron hematoxylin A and B mixture (Electron Microscopy Sciences), followed by PicroSirius Red staining according to the manufacturer’s protocol (Abcam). Stained sections were mounted using Cytoseal XYL (Thermo Scientific) and imaged using Vectra 3.0 Automated Quantitative Pathology Imaging System. For polarized microscopy pictures, Zeiss Axioskop system equipped with a polarizing filter and QICLICK-F-M-12 CCD camera was used.

### Single cell suspension of mouse mammary epithelium and fluorescence-activated cell sorting (FACS)

Mouse mammary glands were harvested, and single cell suspension of the mammary epithelium was prepared as described previously [21, 23]. Briefly, single cell suspension of the mammary glands was enriched for lineage-negative (Lin^−^) epithelial cells using EasySep^TM^ Mouse Epithelial Cell Enrichment kit (Stem Cell Technology) excluding the Lin^+^ cells, specifically hematopoietic (biotinylated CD45 and TER119), endothelial (biotinylated CD31), and immune (biotinylated BP-1) cells. The negatively selected cell population was enriched for luminal epithelium (LE) and mammary stem cell (MaSC)-enriched/basal epithelium (MaSC-enriched). For FACS analysis, Lin^−^ populations were labeled with CD24-PE, CD29-FITC (BD Pharminogen), and CD61-APC (Invitrogen) while their respective isotypes (Thermo Fisher, eBioscience) were used as negative controls. Cell suspensions were incubated with the appropriate antibodies for 30 min on ice. Cells were resuspended in FACS buffer (1 mM EDTA, 1% HI-FBS, and 25 mM HEPES, pH 7.0 in 1× phosphate-buffered saline) and sorted on a FACS BD LSR II Flow Cytometer (BD Biosciences). Data was analyzed using FlowJo software (https://www.flowjo.com).

### RNA isolation and microarray analysis

RNA was isolated using Trizol reagent according to the manufacturer’s protocol (Invitrogen). RNA concentration and quality were assessed using Nanodrop RNA 6000 nano-assays and Bioanalyser. For mouse gene expression analysis, RNA samples were hybridized to Affymetrix GeneChip® Mouse Transcriptome Array 1.0 platform (Affymetrix Inc, Santa Clara, CA) at the Microarray Shared Resource Facility, at The Ohio State University Comprehensive Cancer Center.

### Human gene expression data analysis

To analyze gene enrichment in parous women who breastfed < 6 months vs. ≥ 6 months, we utilized gene expression data obtained from women who were undergoing reduction mammoplasty and were enrolled in the tissue collection study (OSU-2011C0094). Detailed reproductive and other demographic data were also collected at the time of enrollment (Additional file 1: Table S2). Because of the limited number of samples available, we considered the total number of month breastfed for all pregnancies (cumulative no. of months) and did not match for number of pregnancies. Briefly, reduction mammoplasty samples were collected from healthy women and snap frozen in liquid nitrogen. Total RNA isolated from the flash frozen breast tissue was then subjected to gene expression analysis using Human Transcriptome Array 2.0 (Affymetrix Inc, Santa Clara, CA). The affymetrix gene expression data was deposited in NCBI GEO database (GSE102088) [24]. Gene set enrichment analysis (GSEA; http://software.broadinstitute.org/gsea/msigdb/index.jsp) was performed using this published data set [24], querying the C2 curated, hallmark 34 gene sets, and Lim_Luminal_Mammary_Progenitor gene sets (Additional file 1: Table S3) [21] within the Molecular Signatures Database (MSigDB).

### Imaging quantification and scoring

Images of tissue sections harvested from multiple mice (numbers indicated in figure legends) were first captured using Vectra 3.0 and analyzed in inForm®. Routinely, 20 random fields per tissue sections were used for quantification, and all analyses were performed blinded. For the analysis of Ki67, phospho-Stat3(Y705), ERα, and PR, images were segregated into epithelial and stromal compartments, and when needed, into a third compartment for exclusion (i.e., inside the lumen). For trichrome and Picrosirius Red, images were segregated into collagen and non-collagen compartments; for F4/80, CD3, and CD45r, entire images of tissue sections were analyzed. A positive signal threshold for each staining parameter was established before quantification. For quantification of Ki67, F4/80, phospho-Stat3(Y705), ERα, PR, CD3, and CD45r positivity, percent DAB-positive cells in the epithelium, stroma, or both were assessed. For trichrome and PicroSirius Red staining, the total percent positive area was used for quantification. Due to the heterogeneity of ERα stain intensity, we used *H*-score [1 × (% cells 1+) + 2 × (% cells 2+) + 3 × (% cells 3+)] for quantification.

### Statistical analysis

For normally distributed endpoints, or log-transformed data when necessary for variance stabilization, a two-sample *t* test or paired *t* test was used to compare AI vs GI groups. Linear mixed models were used to account for the variance-covariance structure due to repeated measures, such as multiple image data. *p* values were adjusted for multiple comparisons using Holm’s method. The proportion of mice with hyperplastic and metaplastic lesions at day 120 was compared using Fisher’s exact test. GSEA was used to identify pathways and gene sets differentially overrepresented in AI and GI cohorts; false discovery rate (FDR) *q* values were used for multiple comparison adjustment. A *p* value ≤ 0.05 or a *q* value ≤ 0.25 was considered statistically significant. All statistical analyses were performed using SAS/STAT software, v9.2 (SAS Institute) or R3.3.1.

## Results

### Abrupt involution induces histological changes and increased proliferation

Involution following pregnancy and lactation is accompanied by massive cell death and remodeling of the breast, a process that is similar in humans and mice [25, 26]. To address histological changes post-involution, we modeled two types of mammary gland involution, gradual involution (GI) and abrupt involution (AI), in wild type mice of pure FVB/N background (Fig. 1a). Mammary gland morphology determined by whole mount and H&E analyses revealed massive expansion of milk-producing alveoli on postpartum day 7 when all mice were breastfeeding 6 pups (Additional file 1: Figure S1a). All pups were removed from the dams assigned to AI cohort on postpartum day 7. Twenty-one days after removal of all pups in the AI cohort, i.e., on postpartum day 28, the AI glands had remodeled to a near pre-pregnancy state, as evidenced by the absence of glandular secretions within the lumen, and repopulation of stromal adipocytes (Fig. 1b). In contrast, the GI cohort, who were still gradually weaning 6 pups at day 28, exhibited alveolar structures (Fig. 1b) and higher Ki67-positive epithelial cells (*p* = 0.004, Fig. 1e), suggesting continued remodeling of the tissue. Mammary glands of AI and GI mice on day 35 postpartum (4 days after removal of the last 3 pups from the GI cohort) showed alveolar regression and adipocyte repopulation (Fig. 1c). On postpartum day 56 when both GI and AI glands had completed involution, thick fibrous stroma around ducts was present in AI glands only (Fig. 1d). These histological characteristics at day 56 accompanied a twofold increase in epithelial proliferation in AI compared to GI glands (Ki67, *p* = 0.008, Fig. 1e). None of these mice were in diestrous at the time of harvest. Gross anatomical differences between AI and GI glands were not apparent on postpartum day 35 or day 56 (Additional file 1: Figure S1b–d). Comparison of alveolar regression and adipocyte repopulation between GI and AI glands at different time points revealed higher epithelial to stromal ratio in GI glands only at day 28 postpartum as expected (Additional file 1: Figure S1e, 3.5-fold, *p* < 0.001). However, epithelial to stromal ratio was higher in day 35 and day 56 AI glands (Additional file 1: Figure S1e, 1.6- and 2.5-fold respectively, *p* < 0.001).

To address multiparity and determine if abrupt involution after each parity would further modulate these changes, we took females through three rounds of pregnancy with subsequent GI or AI after each pregnancy (Fig. 1f). The mammary glands of stage-matched multiparous mice in the AI cohort exhibited denser ductal lobules (Fig. 1g) and a markedly higher rate of proliferation than those in the GI cohort on day 56 postpartum (6-fold, *p* < 0.0001, Fig. 1h). Here again, the epithelial to stromal ratio was higher in the AI glands (Additional file 1: Figure S1f, 3-fold, *p* < 0.001). These data demonstrate AI following pregnancy results in distinct histological changes and higher proliferation which were not observed in GI glands.

### Abrupt involution induces inflammatory changes

**Fig. 2:**
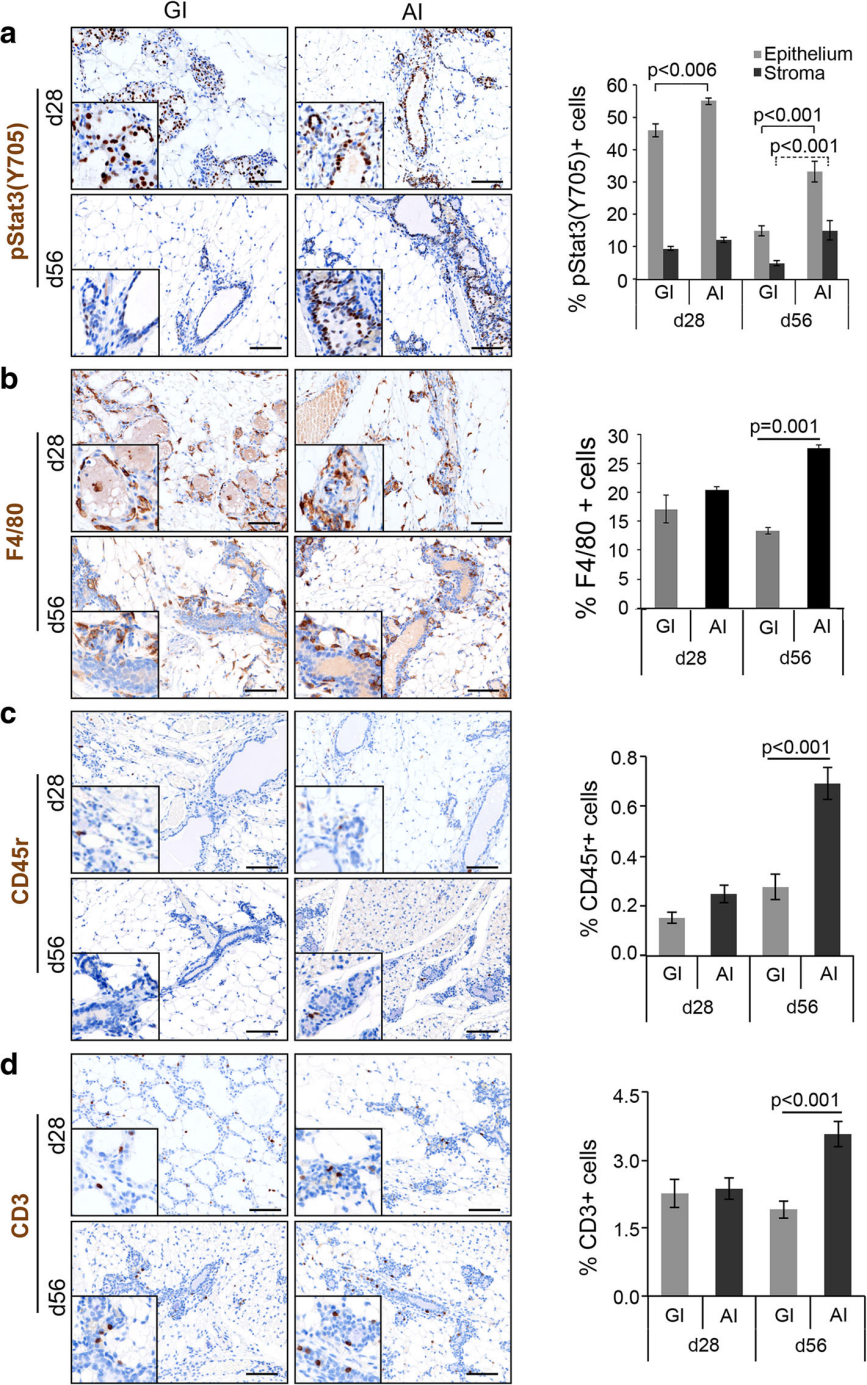
Abruptly involuted glands are highly inflammatory and immune reactive when compared to gradually involuted glands. Representative images of GI and AI mammary gland sections harvested on day 28 and day 56 postpartum and immunostained for **a** pStat3(Y705) and **b** F4/80. Values represented are log transformed. **c** CD45r and **d** CD3 (*n* = 3, scale bar = 100 μM). High-magnification pictures are shown in the inset. The bar diagrams in the respective panel show quantitative difference in expression of the immune markers between the GI and AI glands. Error bars represent standard error of the mean. Linear mixed models were used to calculate significance

The mammary gland upon involution undergoes extensive alveolar cell death, followed by a controlled influx of macrophages and other immune cells for clearance of excess extracellular matrix and phagocytic removal of dead cells, residual milk, and debris [26]. To assess if AI affects macrophage infiltration and immune response differently than GI, we analyzed Stat3 activation and immune cell infiltration. Stat3 is critical for lysosome-mediated programmed cell death during involution [27]. While there was minimal phospho-Stat3 (Y705) in lactating mammary glands (Additional file 1: Figure S2a), on postpartum day 28, we observed a sharp induction of Stat3 phosphorylation in both mammary epithelium and stroma of AI and GI glands (Fig. 2a). Importantly, despite complete involution of AI glands at this time point, we observed small but significantly higher phospho-Stat3 (Y705)-positive epithelial cells in AI compared to GI glands at day 28 (*p* = 0.006, Fig. 2a). By day 56, phospho-Stat3 (Y705) was reduced markedly in GI glands, while it remained elevated in both compartments of the AI glands suggesting a sustained inflammatory state (epithelium: 2.2-fold, stroma: 3-fold, *p* < 0.001, Fig. 2a).

Macrophage (F4/80) infiltration in both GI and AI mammary glands was high and comparable at day 28 postpartum, but it was significantly higher in AI compared to GI glands at day 56 postpartum (2.0-fold, *p* = 0.006, Fig. 2b). Similarly, B cell (CD45r) and T cell (CD3) infiltration was comparable at day 28 postpartum, but significantly higher in AI glands compared to GI glands at day 56 postpartum (2.5-fold, *p* < 0.001 and 1.9-fold, *p* < 0.001, respectively, Fig. 2c, d).

Furthermore, global gene expression analysis of total RNA from GI and AI mammary glands harvested on day 28 postpartum and GSEA analysis querying for hallmark [28] gene sets within the MSigDB revealed negative enrichments of immune-related genes in the GI glands (Additional file 1: Table S1). Negative “normalized enrichment score” for gradual involution reflects upregulation of gene sets in abrupt involution. Among the top 10 gene sets, 4 out of 10 are immune related, all statistically significant (*p* < 0.001) and FDR *q* < 0.003 for all.

In summary, the AI mammary glands display persistent upregulation of inflammatory markers and stromal remodeling consistent with chronic wound healing [29, 30] in contrast to GI mammary glands, which display a transient and tightly regulated immune response with minimal scarring.

### Abrupt involution induces periductal collagen deposition

**Fig. 3:**
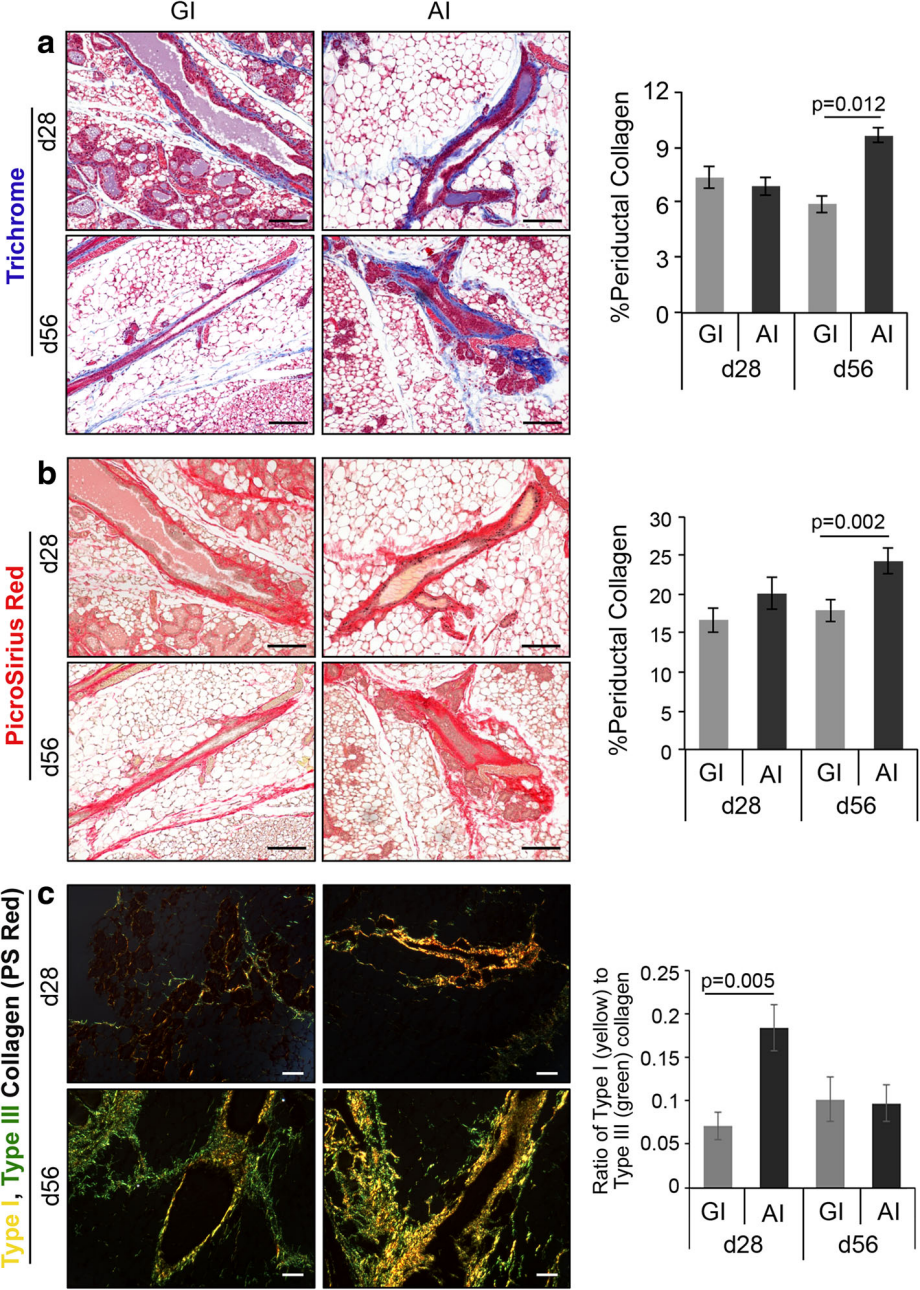
Collagen deposition in mammary glands increases with time following abrupt involution. Representative images of **a** trichrome- (*n* = 3, scale bar = 100 μM) and **b** PicroSirius Red-stained FFPE sections of mammary glands harvested on d28 and d56 postpartum, quantified in the adjacent bar diagrams (*n* = 3, scale bar = 100 μM). **c** Representative images of PicroSirius-stained sections imaged and analyzed for type I (yellow) and type III (green) collagen using polarized light microscopy (*n* = 5 for day28 GI, all other *n* = 6, scale bar = 100 μM). Ratio of type I to type III collagen in the GI and AI glands are quantified in the bar diagram. Error bars represent standard error of the mean. Linear mixed models were used to calculate significance

The mammary stroma or extracellular matrix (ECM) contains different cell types, matrix proteins, and fibers that collectively support mammary development [31]. One of the key components of this ECM is collagen. Higher collagen deposition is associated with a higher risk of developing breast cancer [19, 32]. In particular, deposition of high molecular weight type I collagen has been associated with pro-tumorigenic properties [33]. We performed trichrome staining that revealed a significant increase in collagen deposition in day 56 AI glands compared to matched GI glands (1.7-fold, *p* = 0.012, Fig. 3a), while it was comparable in day 28 glands. PicroSirius Red staining confirmed this observation and showed a similar increase in collagen deposition in AI glands at day 56 (1.4-fold, *p* = 0.002, Fig. 3b). However, additional analysis of PicroSirius Red-stained glands by exploiting its birefringence property [34, 35] revealed the ratio of type I to type III collagen was significantly higher in day 28 AI glands (2.5-fold, *p* = 0.005, Fig. 3c), despite total deposition being comparable to day 28 GI glands. These data demonstrate a distinct pattern of stromal remodeling during AI.

### Abrupt involution increases steroid hormone signaling

**Fig. 4:**
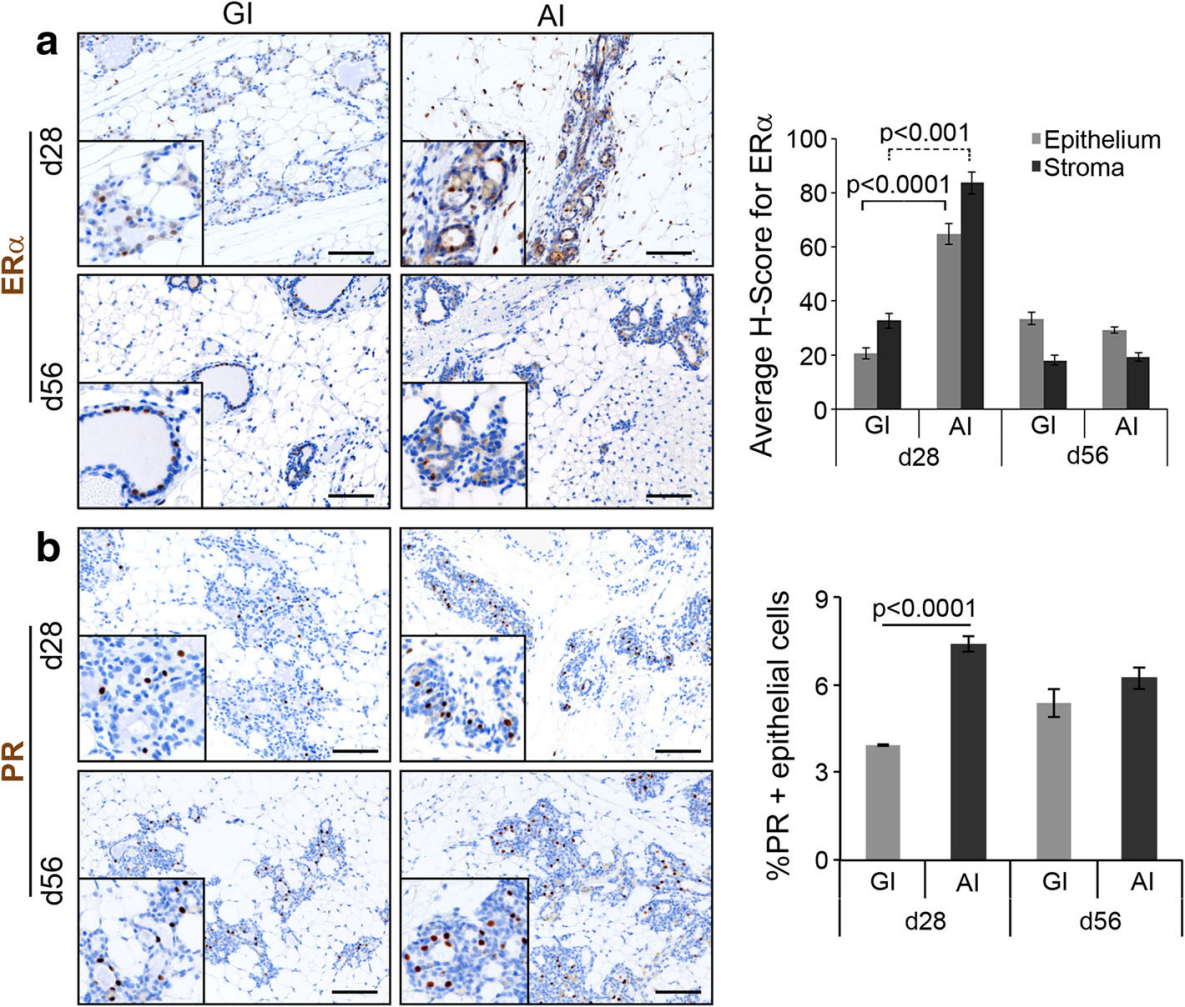
Abruptly involuted glands are exposed to prolonged steroid hormone signaling. **a** Representative images of GI and AI mammary gland sections harvested on day 7 (d7, lactating gland), day 28, and day 56 postpartum and immunostained for ERα. *H*-score of ERα-positive cells in the epithelium and stroma is quantified in the bar diagram (*n* = 3, scale bar = 100 μM). **b** Representative images of GI and AI mammary gland sections harvested on day 7, day 28, and day 56 postpartum and immunostained for PR (*n* = 3, scale bar = 100 μM). Epithelial PR stain is quantified in the bar diagram. Error bars represent standard error of the mean. Linear mixed models were used to calculate significance

The importance of estrogen signaling in mammary gland development and its role in breast cancer have been studied in detail [36, 37]. Here we sought to determine if estrogen signaling was differentially altered due to involution status. Specifically, we assessed levels of ERα and PR in mammary glands by IHC. As shown previously, we observed a moderate level of both epithelial and stromal ERα expression in mammary glands of 10-week virgin nulliparous mice (Additional file 1: Figure S2b) and in lactating glands (Additional file 1: Figure S2d) [38]. On day 28, both epithelia and stroma of AI glands were strongly positive for ERα and significantly higher than those of GI glands (~ 3.0-fold, *p* < 0.001, Fig. 4a), as well as lactating glands. On day 56, the percentage of ERα-positive cells returned to baseline and was comparable between AI and GI glands (Fig. 4a). There was also a 1.9-fold increase in PR-positive epithelial cells in day 28 AI vs GI glands (*p* < 0.0001, Fig. 4b), whereas it was comparable at day 56 (Fig. 4b). Similar to ERα, PR expressing cells were higher in the AI mammary glands at both time points compared to the lactating glands (Additional file 1: Figure S2e). Combined, these data suggest AI glands are subjected to a critical window of higher estrogenic signaling, a known risk factor for developing breast cancer in women [39].

### Abrupt involution results in ductal hyperplasia of the mammary glands

**Fig. 5:**
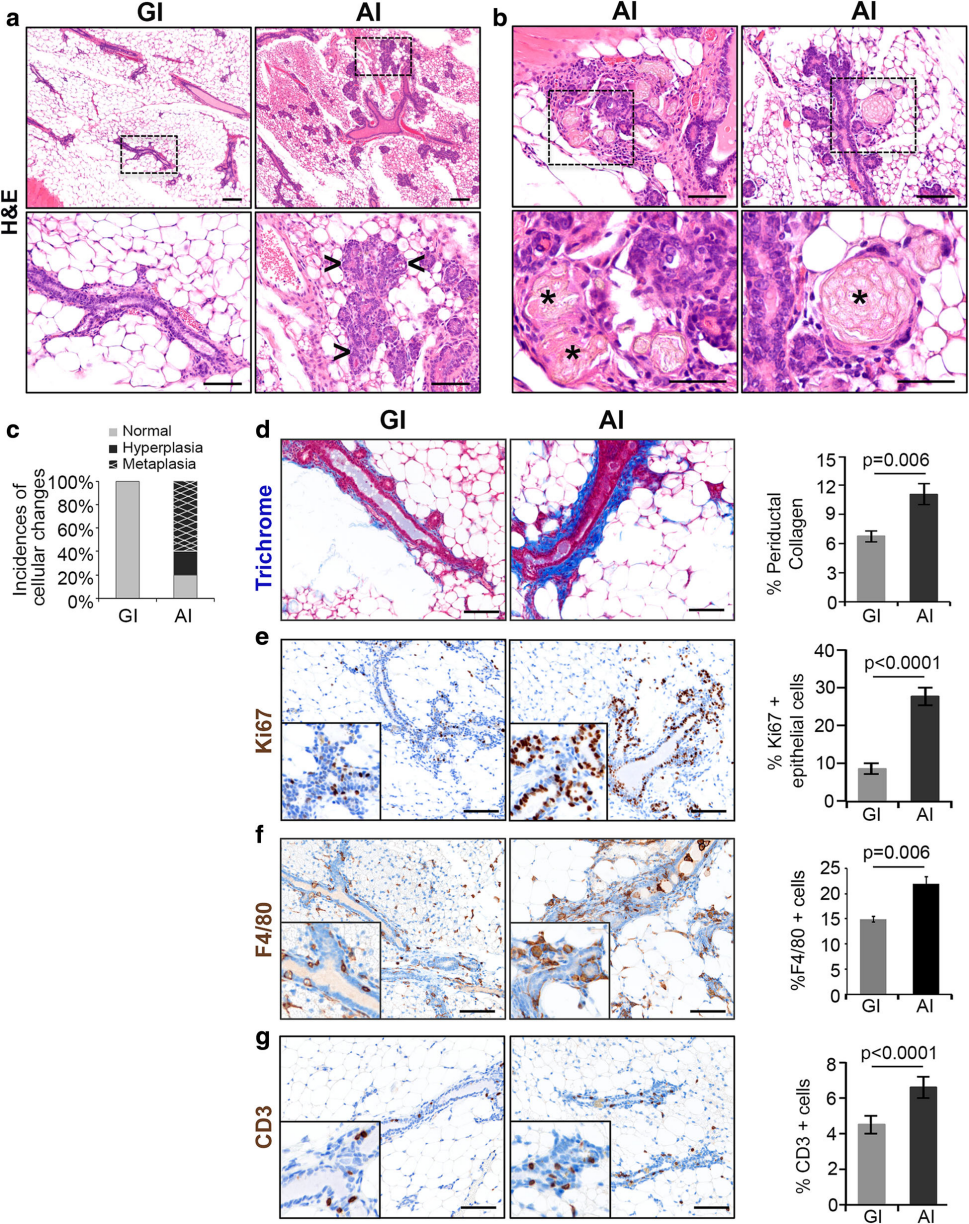
Precancerous changes occur over time in the AI glands. **a** Representative pictures of H&E-stained sections from GI and AI glands harvested on day 120 postpartum (*n* = 5 per group, scale bars = 100 μM, upper panels). The marked area in the upper panel is shown at higher magnification in the lower panels (scale bar = 50 μM). The arrows indicate alveolar hyperplasia. **b** Representative pictures of squamous metaplasia in the AI glands indicated by asterisks (upper panel, *n* = 5, scale bar = 50 μM), magnified in the lower panels (scale bar = 25 μM). **c** Bar diagram showing incidences of hyperplasia (black), metaplasia (hatch), and both (black and hatch) in the GI and AI glands, with normal represented in gray. **d** Representative images of trichrome-stained FFPE sections of GI and AI mammary glands harvested on day 120 postpartum; the percentage of periductal collagen is quantified in the adjacent bar diagram (*n* = 3, scale bars = 100 μM). **e** Representative pictures of Ki67-immunostained sections of mammary glands harvested on day 120 postpartum following GI and AI (*n* = 3, scale bars = 100 μM). Ki67-positive epithelial cells are quantified in the bar diagram. **f** Representative image of GI and AI mammary gland sections harvested on day 120 postpartum and immunostained for F4/80-positive cells (*n* = 3, scale bars = 100 μM) and **g** CD3-positive cells (scale bars = 100 μM). High-magnification pictures are shown in the inset. The bar diagrams show quantitative difference in the expression of the immune markers between the GI and AI glands. Error bars represent standard error of the mean. Linear mixed models were used to calculate significance

To determine the long-term effect of AI on mammary gland morphology, we analyzed GI and AI glands harvested at postpartum day 120. We observed multiple hyperplastic and metaplastic lesions in AI glands, with no such changes observed in the day 120 GI glands (Fig. 5a). Specifically, 4 out of 5 AI mice showed a moderate increase in ductal structures in the mammary glands that was consistent with ductal hyperplasia (*p* = 0.048, Fig. 5a). Three of the five mice showed squamous metaplasia within foci of ductal hyperplasia (*p* = 0.167, Fig. 5b, c). This striking observation was accompanied by an increase in collagen deposition (1.6-fold, *p* = 0.006, Fig. 5d), cell proliferation (3.4-fold, *p* < 0.0001, Fig. 5e), macrophage infiltration (1.4-fold, *p* < 0.001, Fig. 5f), and T cell infiltration (1.75-fold, *p* < 0.001, Fig. 5g) in the AI glands compared to the GI glands.

### Abrupt involution disrupts the mammary epithelial cell hierarchy

**Fig. 6:**
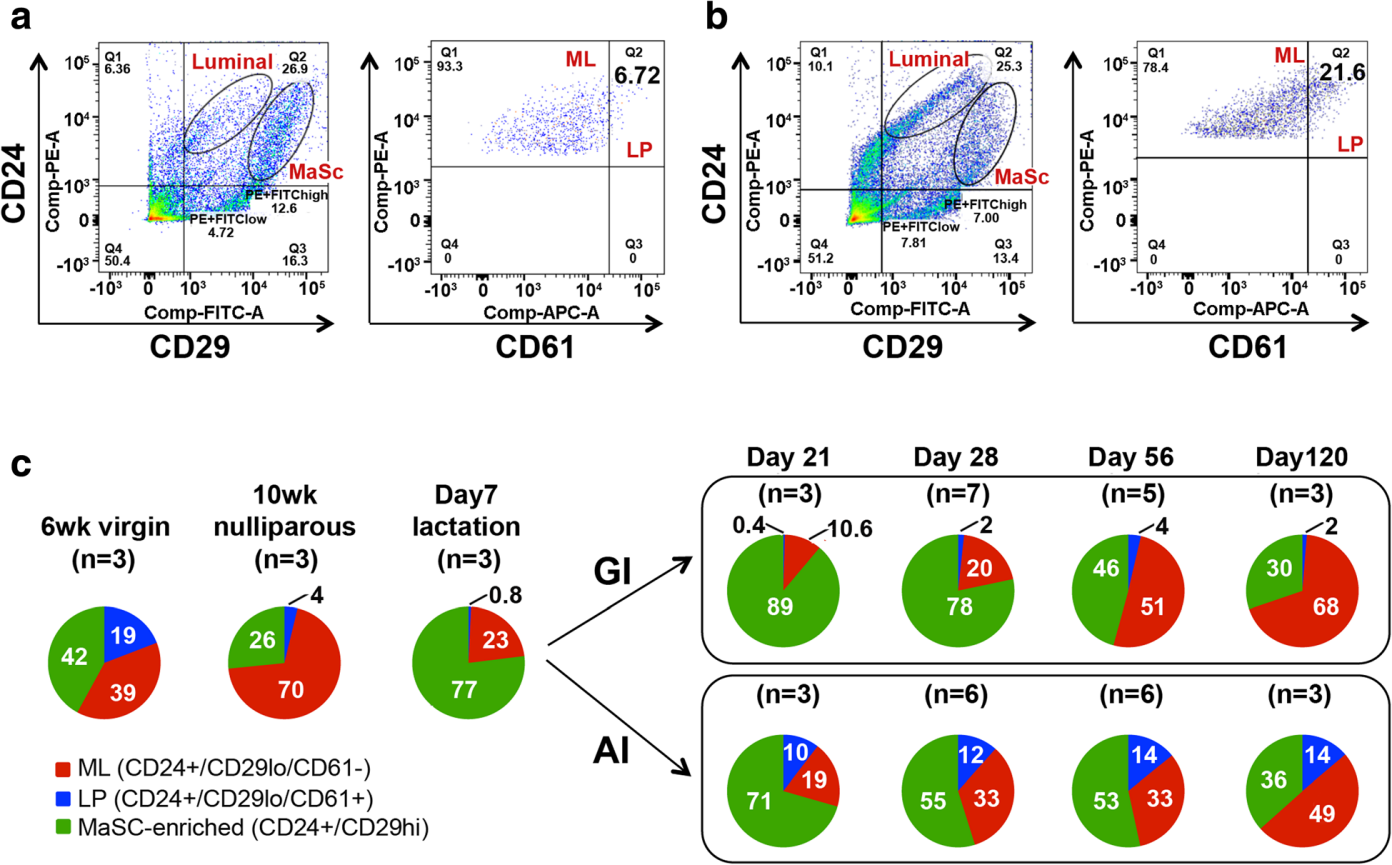
Abrupt involution expands the luminal progenitor cell population and disrupts the epithelial cell hierarchy. FACS analysis of CD24 and CD29 expression in lineage-negative population of the mammary gland (left panel) and CD24 and CD61 expression in the luminal cell population (right panel) in **a** GI and **b** AI mammary gland. **c** Pie chart showing distribution of mammary epithelial subpopulation in 6-week-old virgin mice and 10-week-old nulliparous mice, on lactating day 7 (d7), and on postpartum day 21, day 28, day 56, and day 120 as indicated in the GI and AI cohort. Mammary glands from 6- and 10-week-old virgin mice were used as controls. ML mature luminal cells, LP luminal progenitor cells, MaSC mammary stem cell-enriched population

Given the dysregulated hormone receptor expression in our model and the importance of estrogen signaling in the maintenance of mammary epithelial lineages [40], we hypothesized that epithelial subpopulations may be altered in mice undergoing AI. A hierarchy of stem and progenitor cells is well-defined for mouse mammary epithelium throughout pregnancy, lactation, and involution [41]. Cells committed to luminal lineage were characterized by CD24^+^CD29^lo^ immunophenotype, while cells committed to mammary stem cell (MaSC)-enriched/basal lineage were CD24^+^CD29^hi^ (Fig. 6a, b, left panels). The luminal population was further gated based on expression of CD61 into mature luminal (ML, CD24^+^CD29^lo^CD61^–^) and luminal progenitor (LP, CD24^+^CD29^lo^CD61^+^) subpopulations (Fig. 6a, b right panels, Additional file 1: Figure S3) [21]. Specifically, we analyzed tissue during the following time points: (1) puberty (6 weeks), as the majority of ductal growth during puberty is driven by LP cells [41]; (2) adult virgin nulliparity (10–12 weeks) when ML cells occupy the bulk of glandular epithelia; (3) lactation (day 7 postpartum) when basal/myoepithelial cells predominantly populate the mammary fat pad; and (4) days 21, 28, 56, and 120 postpartum representing different stages of involution and remodeling of the mammary gland.

Strikingly, AI glands interrogated at all four time points postpartum demonstrated a marked expansion of the LP population compared to the GI glands (Fig. 6c). Specifically, there was a 25-fold increase in LP population at day 21 (0.4% GI vs 10% AI), 6-fold increase at day28 (2% GI vs 12% AI), 3.5-fold increase at day 56 (4% GI vs 14% AI), and 7-fold increase at day 120 (2% GI vs 14% AI) (Fig. 6c, Additional file 1: Figure S4). MaSC-enriched population gradually decreased in both cohorts, with the concomitant increase in ML cell compartment. Importantly, the percentage of LP cells in AI glands was comparable to that in the pubertal gland, while the percentage in GI glands was comparable to that of 10-week virgin glands (Additional file 1: Figure S4). These data demonstrate that glands undergoing abrupt, but not gradual remodeling following pregnancy have an altered epithelial composition with a persistent increase in LP cells.

### Notch signaling pathway genes are positively enriched in abruptly involuted mammary tissue obtained from mice and parous women

**Fig. 7:**
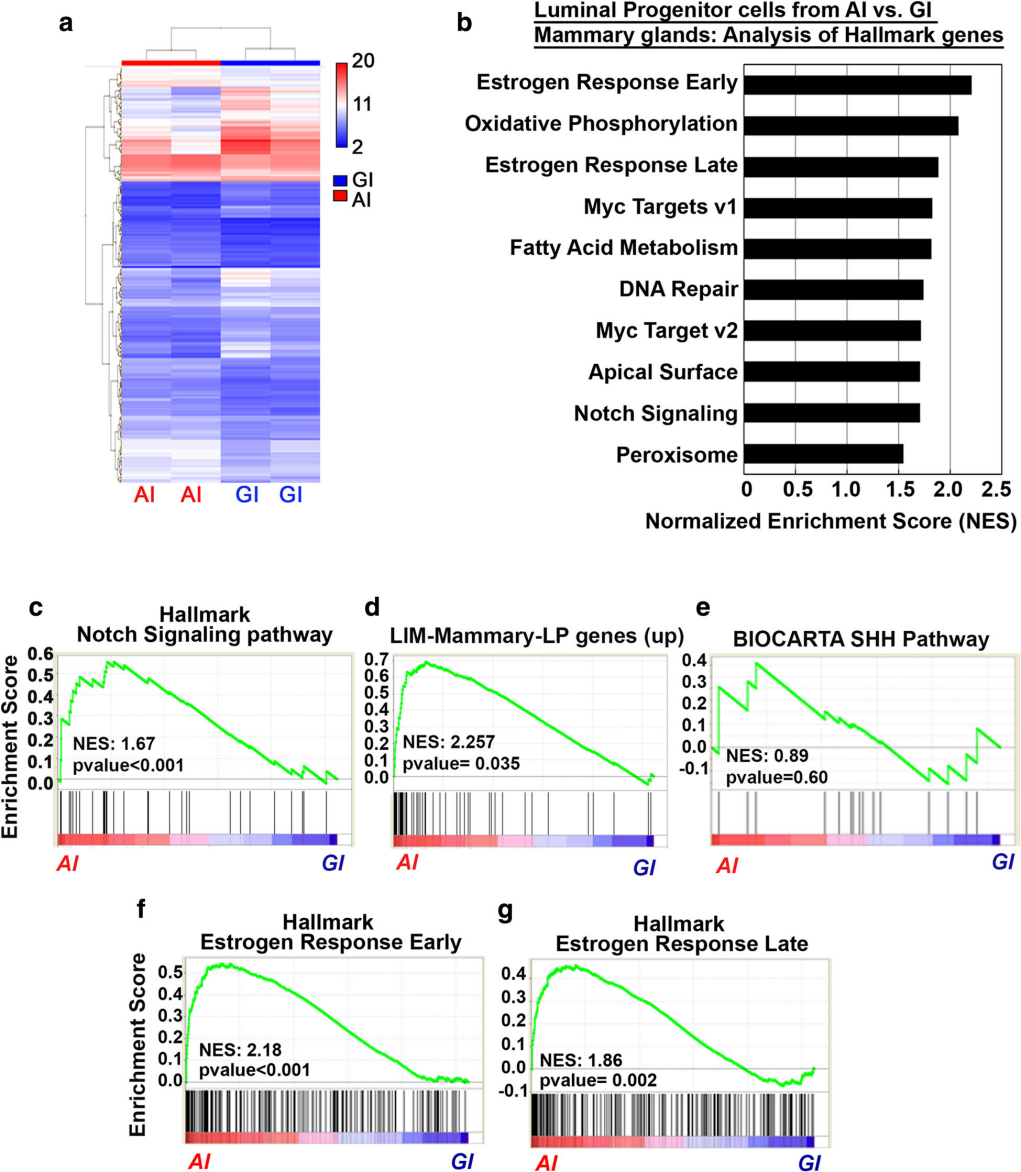
Mouse LP cells harbor gene enrichment for estrogen signaling pathway and Notch signaling pathway. **a** Heatmap showing differential gene expression in mouse mammary LP cells. **b** GSEA querying hallmark genes (h.all.v6.0.symbols.gmt) in mouse mammary LP cells, depicting significant enrichment of **c** estrogen early response genes (HALLMARK_ESTROGEN_RESPONSE_EARLY), **d** estrogen late response genes (HALLMARK_ESTROGEN_RESPONSE_LATE), and **e** Notch signaling pathway genes (HALLMARK_NOTCH_SIGNALING_ PATHWAY). **f** GSEA querying genes consistently up- or downregulated in sorted mammary epithelial cell compartments, showing significant enrichment of **g** genes upregulated in mammary luminal progenitor cells (LIM_MAMMARY_LUMINAL_PROGENITOR_UP) in mouse mammary LP cells isolated from the AI glands when compared to the LP cells from the GI gland. NES normalized enrichment score, FDR false discovery rate, MaSC mammary stem cell, ML mature luminal

To further ascertain the difference in the LP population from AI and GI mice, we analyzed gene expression in sorted LP cells from both glands. Heatmap showing differential gene expression in LP cells isolated from GI and AI glands is shown in Fig. 7a. GSEA querying the C2 curated gene sets and hallmark [28] gene sets within the MSigDB revealed enrichment of several pathways in the AI vs. GI LP cells (Fig. 7b). There was a statistically significant enrichment of Notch pathway genes in AI glands compared to GI glands (NES = 1.67, *p* < 0.001, Fig. 7c). There was also a statistically significant positive enrichment of the known upregulated genes in LP gene signature [21] in the AI glands (NES 2.25, *p* = 0.035, Fig. 7d, Additional file 1: Figure S5). While not significant, a trend in the enrichment of Sonic hedgehog signaling pathway was also observed in mouse LP cells from AI glands (Fig. 7e). Interestingly, hallmark gene set for both estrogen early response genes and estrogen late response genes were enriched in the LP cells from AI glands when compared to those from GI glands (NES = 2.18 and 1.858 respectively, *p* < 0.001, Fig. 7f, g). This data demonstrates that increased expression of ERα and PR in day 28 AI glands led to an increase in estrogen signaling.

**Fig. 8:**
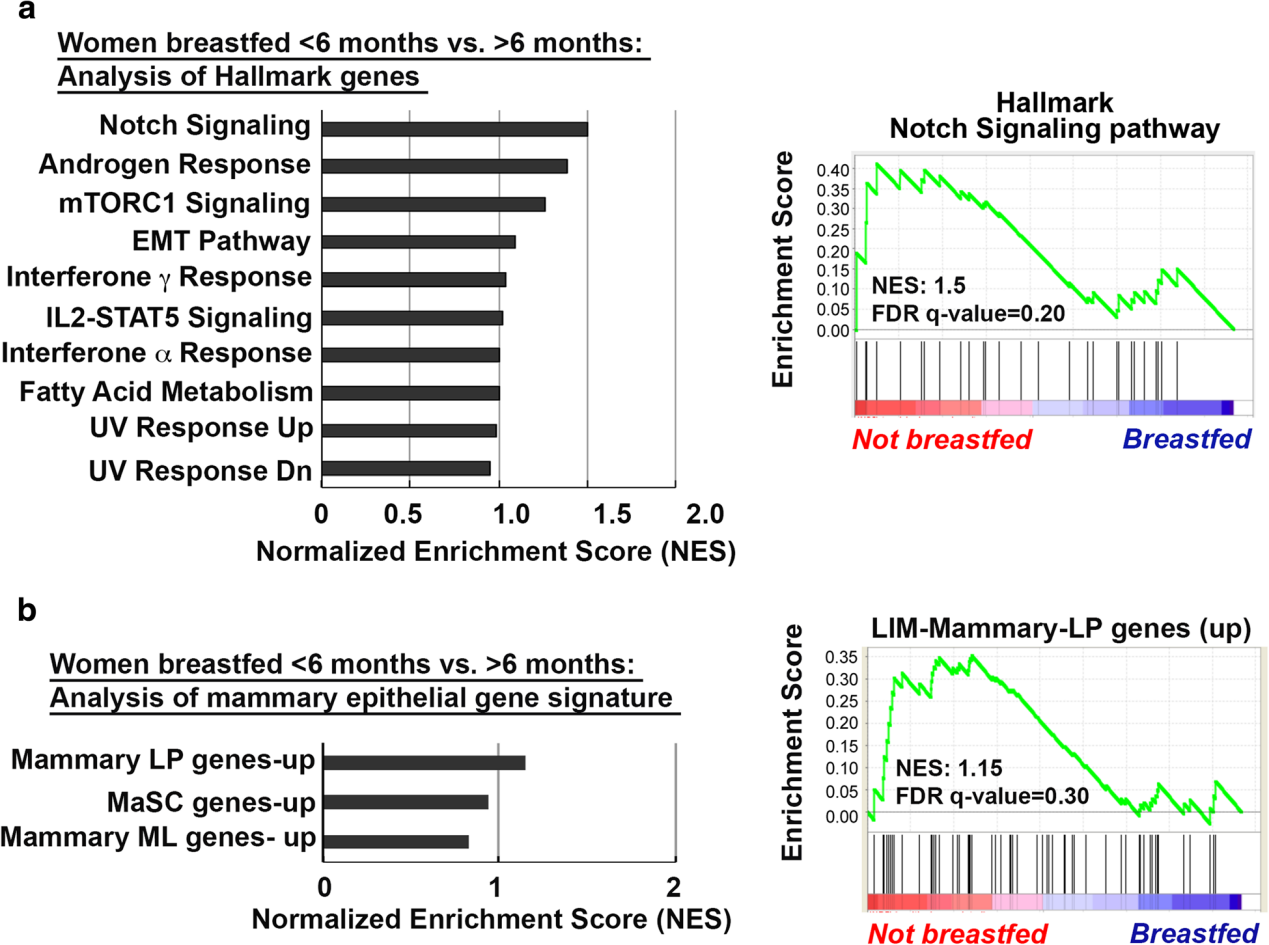
Human breast tissue from women who breastfed < 6 months harbors gene enrichment for Notch signaling pathway. **a** GSEA querying hallmark genes (h.all.v6.0.symbols.gmt) (left panel) depicting significant enrichment of Notch signaling pathway genes (HALLMARK_NOTCH_SIGNALING_PATHWAY, right panel). **b** GSEA querying genes consistently up- or downregulated in sorted mammary epithelial cell compartments (left panel), showing significant enrichment of genes upregulated in mammary luminal progenitor cells (LIM_MAMMARY_LUMINAL_PROGENITOR_UP, right panel) in breast tissue of women who breastfed for < 6 months, when compared to women who breastfed ≥ 6 months. NES normalized enrichment score, FDR false discovery rate

To determine if the gene expression changes observed in our mouse model are recapitulated in parous women who did not breastfeed, we compared gene expression in normal breast tissue obtained from premenopausal women with no history of breast cancer who breastfed for ≥ 6 months (GI, *n* = 16) versus those who breastfed for < 6 months (AI, *n* = 16) (Additional file 1: Figure S6). Samples were obtained from women enrolled in an IRB-approved tissue collection protocol to undergo reduction mammoplasty who consented to provide tissue for research. Detailed reproductive and other demographic data were also collected at the time of enrollment (OSU-2011C0094) (Additional file 1: Table S2). This time frame of breastfeeding was selected based on epidemiological studies showing benefit from at least 6 months of breastfeeding [2, 4, 7, 8]. Within this data, GSEA revealed a strong positive enrichment for Notch signaling pathways in women who breastfed for < 6 months when compared to those who breastfed for ≥ 6 months (Fig. 8a). The FDR *q* values for this pathway was 0.20 and *p* value was 0.039. This finding illustrates that in our cohort more than 3 in 4 women who breastfed for < 6 months share this phenotype. Furthermore, women who breastfed for < 6 months demonstrated a trend towards positive enrichment of LP gene signature (Fig. 8b), FDR *q* value = 0.30, *p* value = 0.3. Taken together, our data suggests that breastfeeding for less than 6 months is associated with distinct molecular changes in breast tissue, i.e., enrichment for stem cell self-renewal and growth signaling pathways.

## Discussion

Population studies provide extensive evidence that among parous women, prolonged breastfeeding protects against the risk of developing breast cancer, particularly TN/BLBC [2, 9, 17, 42]. The large collaborative analysis of data from 47 epidemiological studies from 30 countries including 50,302 women with breast cancer and 96,973 controls concluded that for every 12 months of lifetime duration of breastfeeding, breast cancer risk reduces by 4.3% (95% CI 2.9–5.8; *p* < 0.0001) [1]. A more recent meta-analysis of 32 studies done in 2013 showed that parous women who breastfed had a 14% reduced risk of developing breast cancer when compared to parous women who did not breastfeed and this was even greater (28%) for women who breastfeed for over 12 months cumulatively irrespective of number of births [43]. While it has long been believed that parity reduces risk of breast cancer, recent evidence suggests that this is primarily for ER-positive subtype [44]. For TNBC, parity may actually increase the risk of developing cancer, but this risk can be modulated by breastfeeding [2, 7, 45–48]. In a case series study, parous women who never breastfed had a risk of developing TNBC that was 2.18 times (95% CI 1.52, 3.12) higher than nulliparous women [48]. In addition, the results from AMBER consortium study showed that among parous women, the risk of developing ER-negative breast cancer increased markedly by increasing parity among women who never breastfed (odds ratio 1.22 for one birth vs 1.68 for four or more births) [7]. However, to date, the morphological changes associated with gradual involution occurring after prolonged breastfeeding and how it protects from breast cancer, particularly TNBC, is not well-elucidated.

Pioneering work studying the mechanism underlying mammary gland involution and its association with breast cancer used mouse models in which pups were weaned abruptly on postpartum day 10 [19, 20]. A detailed mechanism of events following abrupt involution has been elucidated using this model. We followed the same weaning strategy to induce abrupt involution, as pups were removed abruptly while the dam was actively nursing the pups. This model mimics a situation of abrupt involution in humans (e.g., return to work, illness), whereby the breast has undergone alveologenesis to produce milk but following birth, breastfeeding was stopped abruptly within a short time. Our study is novel in that it compared the effects of abrupt involution of mammary glands with gradual involution that occurs following prolonged breastfeeding. For most strains of mice, pups start feeding on solid food typically around 16 days and are weaned by day 21. In order to ensure uniformity of our prolonged lactation, we ensured that each dam was kept with 6 pups in separate cages. In addition, we weaned the pups three at a time at day 28 and day 31 to produce the effect of gradual weaning. We have developed this model to mimic human breastfeeding patterns based on duration and weaning with or without a preceding gradual decline in breastfeeding (GI vs AI). We report here the distinctly variable impact of AI vs GI on the mammary glands of FVB/N mice that could alter the future risk of developing breast cancer.

Increased stromal collagen, particularly type I collagen, is associated with regions of high breast density [49] and is one of the strongest independent risk factors for developing breast cancer [50–52]. AI resulted in increased collagen deposition in mouse mammary glands, with higher type I to type III collagen ratio, suggesting that duration of breastfeeding following each pregnancy could impact mammographic density. Elegant studies have shown increased type I collagen deposition in involuting glands and have been implicated as mediators of inflammation and macrophage recruitment [53–55]. Higher collagen density is also associated with increased proliferation of the mammary epithelium, ERα receptor expression, and function [32, 56]. Current evidence shows that persistent ERα signaling is critical for the development of ERα-negative breast cancers [57] and that ERα/PR-positive luminal epithelial cells play a key role in the expansion of ERα/PR-negative progenitor cells [58–60], the putative cell of origin for BLBC [21]. Our observation of higher collagen deposition, increased expression of ERα, and proliferation index in AI glands when compared to GI glands suggests that lack of or short-term breastfeeding can lead to a pro-tumorigenic environment. Furthermore, on longer follow-up at day 120, AI glands developed hyperplasia and squamous metaplasia. This is a very interesting finding supporting the link between lack of breastfeeding and higher risk of developing breast cancer as ductal hyperplasia is a well-known non-obligate precursor of human breast cancer [61]. Multiple studies have shown that evolution of breast carcinogenesis is a multi-step process by which intermediate hyperplastic lesions, such as usual hyperplasia undergo various grades of atypia and develop into atypical ductal hyperplasia, ductal carcinoma in situ, and invasive cancer. Current literature demonstrates usual ductal hyperplasia increases breast cancer risk by 1.5- and 2-fold [61, 62]. This adds a new dimension to the conventional thinking that postpartum mammary gland involution is highly tumorigenic [63–65] and instead demonstrates that GI may confer protection against a tumorigenic and pro-inflammatory environment seen in acutely remodeling mammary glands. In fact, GSEA analysis of global gene expression revealed negative enrichment of immune-related pathways in the GI glands supporting the protective effect of prolonged breastfeeding. We plan to confirm our findings through the identification of specific signaling pathways that are activated only in the AI glands leading to the pro-tumorigenic environment. Furthermore, we plan to expose the AI mice to anti-inflammatory agents to determine if these pathways are downregulated and abrogate the hyperplastic changes.

Analysis of the epithelial cell hierarchy revealed a clear and persistent expansion of mammary LP cells in AI glands. This is an important observation, as BLBC associated with *BRCA1* mutations [16, 66] shows similar lineage commitment defects that occur prior to tumorigenesis [67]. Furthermore, a similar increase in the LP population has been reported in healthy *BRCA1* mutation carriers, who are at high risk of developing BLBC [21].

Our findings demonstrated that breast tissue from healthy, premenopausal women who breastfed for < 6 months showed a positive enrichment of genes within Notch signaling pathways that regulate mammary stem cell function and luminal cell-fate commitment [68]. This observation lends further evidence that lack of or short duration of breastfeeding may drive LP expansion, supported by the strong trend towards enrichment in the LP gene signature seen in this cohort of women.

A potential limitation to this study is the small sample size for gene expression analysis from breast tissue and variability within each group with respect to time elapsed since breastfeeding and tissue sampling, despite endpoints meeting statistical significance. Although the two groups (breastfed < 6 months vs ≥ 6 months) were well-matched with respect to age, ethnicity, age at menarche, and age at first birth, we acknowledge that the distribution of BMI was not well-balanced, and this may have impacted the findings. Further studies are underway to obtain normal breast tissue from parous women with known breastfeeding history and controlled for all parameters. Another caveat is that although we show distinct histological features that are potentially precancerous in the abruptly involuted glands, we do not report invasive tumor development. Our goal was to determine how short-term and prolonged breastfeeding differentially impacted the breast microenvironment that alters the future risk of developing breast cancer. Future studies will address the development of invasive cancer, using our models of involution.

Taken together, we have shown that aberrant expansion of LP cells with increased stemness in an inflammatory milieu can lead to a pro-tumorigenic environment in the AI glands. This, associated with an increase in proliferation observed in AI glands along with ductal hyperplasia, increases the likelihood of developing somatic mutations over time (such as loss of *BRCA1*, *Pten*, and/or *p53*) that could ultimately give rise to basal-like malignancy. Our data showing hyperplastic lesions only in the abruptly involuted and not in the gradually involuted glands supports the epidemiological data that breastfeeding impacts the risk of developing breast cancer.

## Conclusions

Prolonged breastfeeding can help reduce the risk of developing breast cancer including aggressive subtypes. Our preclinical work provides novel insight into the known link between breastfeeding and breast cancer. We observed distinct pro-tumorigenic changes in mouse mammary glands that underwent abrupt involution that was not observed in the gradually involuting glands demonstrating the potential protective effects of prolonged breastfeeding in parous women. Our findings can therefore help strengthen the efforts in promoting the initiation and maintenance of breastfeeding for at least 6 months [69, 70]. Further delineation of this mechanistic link in the future will help to develop prevention measures for mothers who are unable to breastfeed.

## Additional file

Additional file 1: This file contains **Figure S1–S6** and **Tables S1–S3.** (DOCX 1111 kb)

## Data Availability

All experimental data are provided in the manuscript.

## Abbreviations

AI: Abrupt involution
BLBC: Basal-like breast cancer
ER: Estrogen receptor
FACS: Fluorescence-activated cell sorting
FDR: False discovery rate
GI: Gradual involution
GSEA: Gene set enrichment analysis
LP: Luminal progenitor
MASC: Mammary stem cell
ML: Mature luminal
MSigDB: Molecular Signature Database
NES: Normalized enrichment score
PR: Progesterone receptor
TNBC: Triple-negative breast cancer
