# Supplementary material for: Abrupt involution induces inflammation, estrogenic signaling, and hyperplasia linking lack of breastfeeding with increased risk of breast cancer
Source: Breast Cancer Res. 2019 Jul 17;21:80. doi: 10.1186/s13058-019-1163-7 (PMC6637535; doi:10.1186/s13058-019-1163-7)
Supplement: Supplementary file 1 — This file contains Figure S1–S6 and Tables S1–S3. (DOCX 1111 kb) [file 13058_2019_1163_MOESM1_ESM.docx]

**Supplementary Figures**


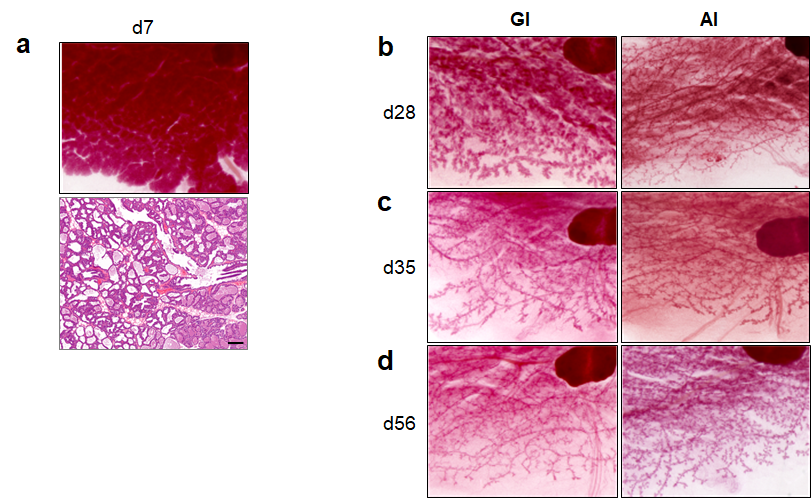


**
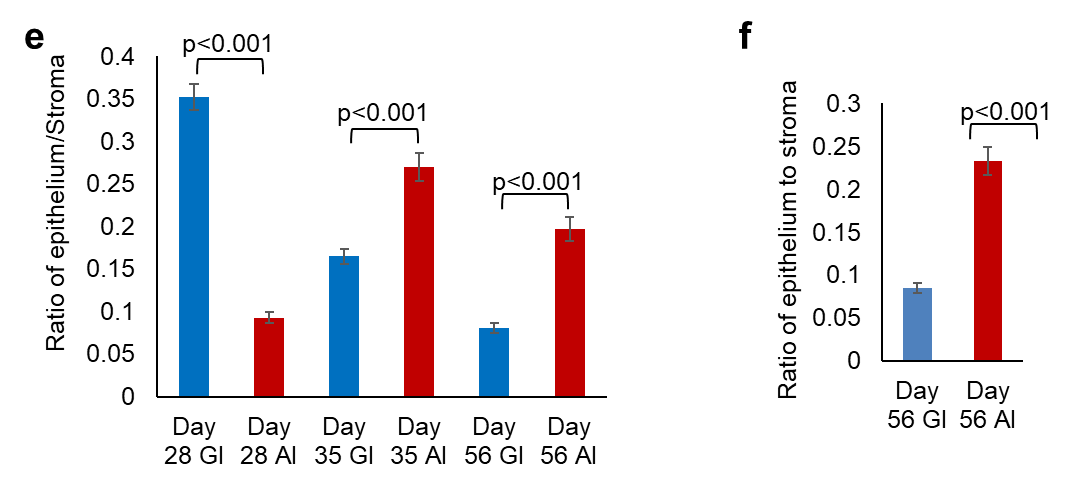
**

**Additional file 1: Figure S1: a.** Representative images of lactating mammary gland on day 7, whole mount (upper panel), H&E stained FFPE section (lower panel, scale bar 100μM). **b.** Representative images of mammary gland whole mounts harvested from GI and AI mice on day 28, **c.** day 35 and **d.** day 56 postpartum. **e.** Ratio of epithelium to stroma in GI and AI mammary glands at indicated time points following single parity and **f.** multiparity (n=3).

**
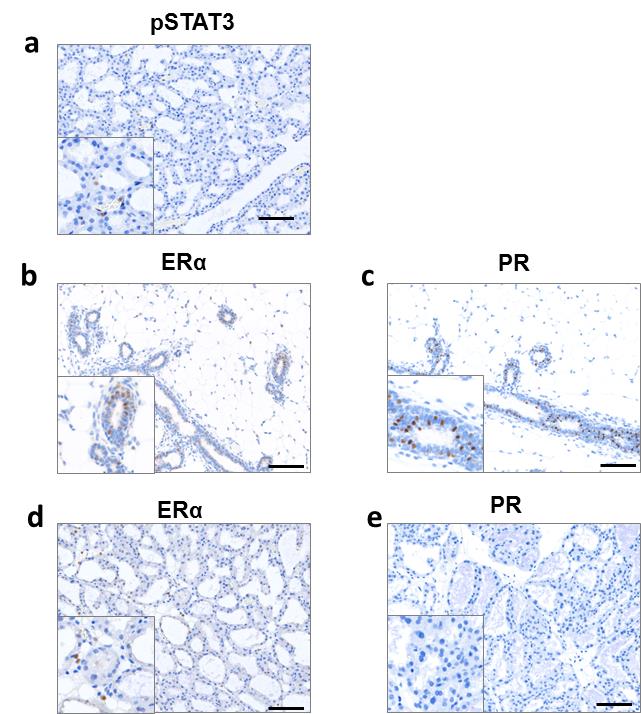
**

**Additional file 1: Figure S2:** Representative images of mammary glands harvested from **a.** lactating mice (day 7 postpartum) immunostained for pStat3(Y705), **b.** 10 week old virgin nulliparous mice immunostained for ERα and **c.** PR. (n=3 mice/group, scale bar=100μM). **d.** lactating mice (day 7 postpartum) immunostained for ERα and **e.** PR. (n=3 mice/group, scale bar=100μM).


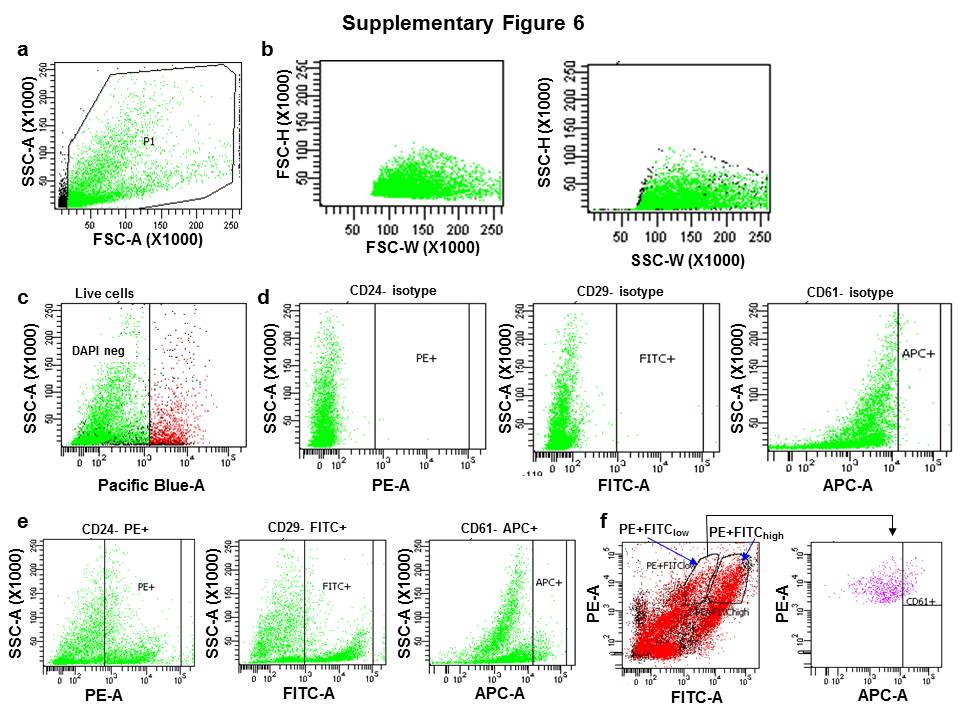


**Additional file 1: Figure S3: Gating strategy for FACS analysis. a.** Strategy for identification of live cells **b.** Exclusion strategy for doublets **c.** Cells stained with DAPI and gated for DAPI negative (live cells) **d.** Isotype controls for CD24, CD29, and CD61 **e.** Single antibody positive controls for CD-24-PE, CD29-FITC, CD61-APC, and **f.** Gating strategy to determine stem cell populations. Cells co-stained with CD-24-PE, CD29-FITC, CD61-APC were sorted with the gating as shown for CD24+CD29-low, CD24+ CD29High cell populations (left panel). CD24+CD29 low cell population was further gated for CD61 positivity (right panel).


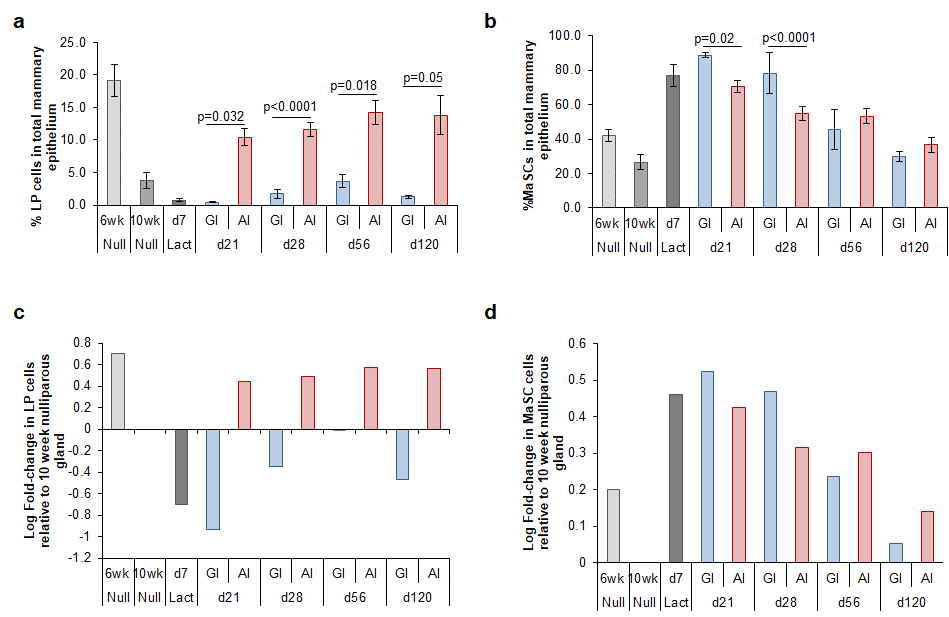


**Additional file 1: Figure S4: a.** Comparison of LP cell population and **b.** MaSC population in the mammary gland harvested at indicated time points. **c.** Fold change in LP population and **d.** fold change in MaSC population compared to 10 week old virgin nulliparous gland. Two sample *t*-test was used to assess significance for each time point with Holm’s correction for significance.


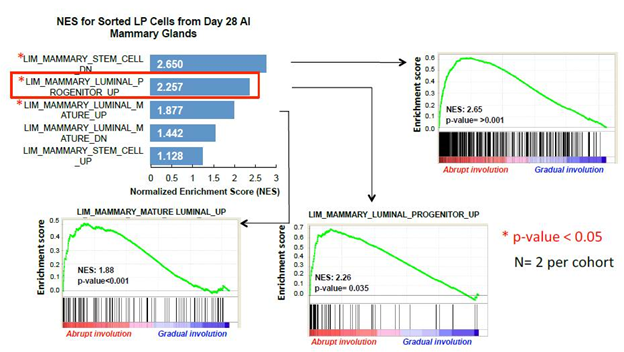


**Additional file 1: Figure S5:** GSEA querying genes consistently up- or downregulated in sorted mouse mammary luminal progenitor cells.


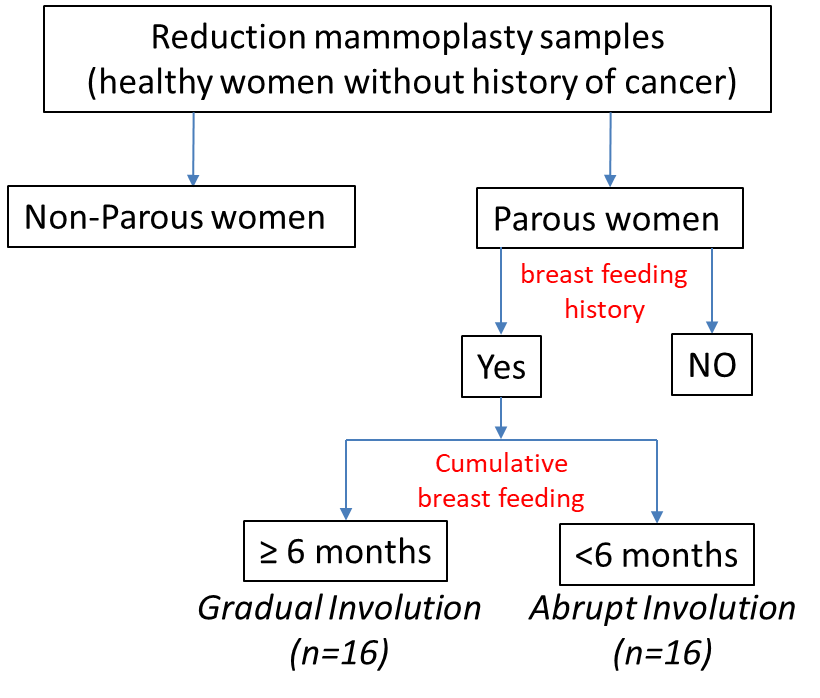


**Additional file 1: Figure S6.** Flow chart showing sample selection for GI and AI cohort, which was followed by analysis of the gene expression data (GSE 102088).

**Additional file 1: Table S1.** Signatures enriched in Gradual Involution relative to Abrupt Involution.

|  | Enrichment Score | Normalized Enrichment Score | NOM  p-value | FDR  q-value | FWER p-value | Immune Related |
| --- | --- | --- | --- | --- | --- | --- |
| HALLMARK_INTERFERON_  ALPHA_RESPONSE | -0.583 | -2.155 | 0 | 0 | 0 | **Yes** |
| HALLMARK_INTERFERON_  GAMMA_RESPONSE | -0.525 | -2.125 | 0 | 0 | 0 | **Yes** |
| HALLMARK_MITOTIC_  SPINDLE | -0.494 | -2.021 | 0 | 4.4E-04 | 0.001 | No |
| HALLMARK_IL6_JAK_  STAT3_SIGNALING | -0.539 | -1.935 | 0.002 | 0.00157 | 0.005 | **Yes** |
| HALLMARK_PROTEIN_  SECRETION | -0.524 | -1.923 | 0 | 0.00213 | 0.008 | No |
| HALLMARK_P53_  PATHWAY | -0.462 | -1.881 | 0 | 0.00275 | 0.012 | No |
| HALLMARK_ESTROGEN_  RESPONSE_LATE | -0.457 | -1.862 | 0 | 0.00307 | 0.016 | No |
| HALLMARK_G2M_  CHECKPOINT | -0.446 | -1.823 | 0 | 0.00300 | 0.018 | No |
| HALLMARK_APOPTOSIS | -0.458 | -1.799 | 0 | 0.00322 | 0.022 | No |
| HALLMARK_  INFLAMMATORY_  RESPONSE | -0.438 | -1.795 | 0 | 0.00289 | 0.022 | **Yes** |

**Additional file 1: Table S2.** Characteristics of the study population enrolled in OSU-2011C0094 and included in the gene expression analysis.

|  | Breastfeeding  ≤6 months | Breastfeeding >6months |
| --- | --- | --- |
| Total number | 16 | 16 |
| Race |  |  |
| Caucasian | 11 | 12 |
| African-American | 5 | 4 |
| BMI |  |  |
| <25 | 0 | 7 |
| ≥25 to <30 | 7 | 3 |
| ≥30 to <40 | 5 | 6 |
| ≥40 | 4 | 0 |
| Age of Menarche |  |  |
| <13 | 4 | 5 |
| ≥13 to <15 | 9 | 8 |
| ≥15 | 3 | 3 |
| Parity |  |  |
| 1 | 9 | 2 |
| 2 | 5 | 7 |
| 3+ | 2 | 7 |
| Age, First Birth |  |  |
| <20 | 3 | 1 |
| ≥20 to <30 | 7 | 5 |
| ≥30+ | 3 | 5 |
| Unknown | 3 | 4 |
| Age, Last Birth |  |  |
| <20 | 1 | 0 |
| ≥20 to <30 | 9 | 1 |
| ≥30+ | 3 | 11 |
| Unknown | 3 | 4 |
| Breastfeeding, Total |  |  |
| <4 months | 13 | 0 |
| ≥4 to <6 months | 3 | 0 |
| ≥6 months to <1 year | 0 | 6 |
| ≥1 to <2 years | 0 | 4 |
| ≥2 years | 0 | 6 |

**Additional file 1: Table S3.** GSEA querying genes consistently up- or downregulated in sorted mammary epithelial cells both in mouse and human cohorts.

| **Gene Set Queried [19]** | **Description** |
| --- | --- |
| LIM_MAMMARY_LUMINAL_MATURE_DN | Genes consistently down-regulated in mature mammary luminal cells both in mouse and human species. |
| LIM_MAMMARY_LUMINAL_MATURE_UP | Genes consistently up-regulated in mature mammary luminal cells both in mouse and human species. |
| LIM_MAMMARY_LUMINAL_PROGENITOR_DN | Genes consistently down-regulated in mammary luminal progenitor cells both in mouse and human species. |
| LIM_MAMMARY_LUMINAL_PROGENITOR_UP | Genes consistently up-regulated in mammary luminal progenitor cells both in mouse and human species. |
| LIM_MAMMARY_STEM_CELL_DN | Genes consistently down-regulated in mammary stem cells both in mouse and human species. |
| LIM_MAMMARY_STEM_CELL_UP | Genes consistently up-regulated in mammary stem cells both in mouse and human species. |
